# Radioactive iodine and male reproductive health in thyroid cancer survivors: evidence of delayed gonadal dysfunction

**DOI:** 10.1007/s40618-025-02725-y

**Published:** 2025-10-15

**Authors:** Daniele Santi, Giorgia Spaggiari, Antonio R.M. Granata, Tommaso Piticchio, Donatella Paoli, Francesco Lombardo, Manuela Simoni, Francesco Pallotti

**Affiliations:** 1 Unit of Endocrinology, Department of Biomedical, Metabolic and Neural Sciences, University of Modena and Reggio Emilia, Via Giardini 1355, Modena, 41126 Italy; 2 Unit of Andrology of the Unit of Endocrinology, Department of Medical Specialties, Azienda Ospedaliero-Universitaria of Modena, Modena, Italy; 3 Department of Biomedical, Metabolic and Neural Sciences, University of Modena and Reggio Emilia, Modena, Italy; 4 Department of Medicine and Surgery, Kore University of Enna, Enna, 94100 Italy; 5 Laboratory of Seminology, Sperm Bank “Loredana Gandini”, Department of Experimental Medicine, Sapienza University of Rome, Rome, Italy

**Keywords:** FSH, RAI, Radioactive iodine, Thyroid carcinoma, Male infertility

## Abstract

**Purpose:**

Differentiated thyroid carcinoma (DTC) incidence is rising globally, with a higher prevalence in women. Male patients often present with more aggressive disease, leading to more frequent use of radioactive iodine (RAI) therapy. While the gonadotoxic effects of RAI in females have been studied, its impact on male reproductive health remains unclear. This study is aimed to assess the effects of RAI therapy on gonadal function in men with DTC, focusing primarily on follicle-stimulating hormone (FSH) serum levels as a biomarker of testicular function.

**Methods:**

We analyzed studies reporting FSH levels before and after RAI administration in male DTC patients. The primary outcome was the change in FSH levels over time, with secondary outcomes including luteinizing hormone (LH) levels and semen parameters.

**Results:**

Seven studies comprising 460 men met inclusion criteria. FSH levels significantly increased 12 months post-RAI (mean difference6.56 IU/L; *p* < 0.001), but not at 3 or 6 months. Meta-regression revealed that FSH elevation was positively associated with patient age and RAI dosage. No significant changes were observed in LH levels or standard semen parameters. Despite high heterogeneity (I²=99%), sensitivity analyses confirmed the findings.

**Conclusion:**

RAI therapy in male DTC patients is associated with delayed and dose-dependent increases in FSH, suggesting a potential subclinical impact on testicular function, particularly spermatogenesis. Standard semen parameters remain stable initially, possibly due to compensatory mechanisms. Powerful, prospective studies are needed to assess long-term reproductive outcomes and explore qualitative sperm alterations, including DNA integrity, to better inform fertility counseling in male DTC patients undergoing RAI.

## Introduction

Differentiated thyroid carcinoma (DTC) is among the fastest-growing human malignancies worldwide, with an estimated incidence of 5–10 new cases per 100,000 people annually [1]. DTC is significantly more common in women than men, with an age-adjusted incidence of 13.3 per 100,000 in females and 4.9 per 100,000 in males [2, 3]. However, these reports highlighted a more advanced disease in men compared to women. Indeed, male patients had larger tumors, higher rates of lymph node involvement (33.2% vs. 21.0%), and more distant metastasis (2.3% vs. 0.9%) compared to female patients [2, 3]. Moreover, a large proportion of DTC cases are expected in younger individuals, with an estimated 65% of DTC cases are diagnosed in populations under 55 years [4]. Despite its rising frequency, DTC generally has an excellent prognosis, with 5-year survival rates of 99.9% for localized disease and 74.2% for distant metastases. The expanding patient population underscores both the need to optimize management strategies and to look for long term effects of RAI treatments [5]. According to scientific guidelines, the gold standard treatment for DTC remains surgical thyroid gland removal, with the specific approach varying based on tumor and patient characteristics [6–8]. Radioactive iodine (RAI) administration, using iodine-131 (I-131), plays a pivotal role in the post-operative management of DTC [9, 10], at least for patients with intermediate/high risk cancer at the diagnosis. Many studies are available in the literature, showing that RAI therapy can significantly enhance long-term survival rates or reduce risk of recurrence [11–15].

Despite the known efficacy of RAI on cancer recurrence and patient’s survival, potential adverse effects of RAI on different physiological systems remain a concern. Among most relevant consequences of RAI, lung injury and potential second primary tumor occurrence have been hypothesized [16–18]. Moreover, the potential negative impact of RAI on the reproductive system has been largely advocated, at least in female subjects [19, 20]. This represents a milestone in the clinical management of female oncology patients. Guidelines also recommend discussing family planning with female patients before RAI treatment, particularly informing women over 35 about its potential impact on pregnancy achievement [20].

Conversely, how we should address male patients’ questions regarding the potential impact of RAI on their fertility is still not clear. Although DTC is less common in men, it often presents with a more aggressive clinical course at diagnosis and is associated with a poorer prognosis [21, 22]. As a result, RAI is more frequently selected as part of post-operative management in male patients with DTC, likely with more frequent administration of adjuvant doses. Thus, the potential impact of RAI on male reproductive health should be more detailed and studied [23]. Since the testicular germinal epithelium, especially spermatogonial stem cells, is highly susceptible to radiation damage [24–26], exposure to I-131 may pose risks to testicular function. Concerns have been raised regarding whether RAI could cause temporary or lasting impairments in spermatogenesis and hormone production. While several studies have explored these effects, the findings remain inconclusive due to variations in research methodologies, patient monitoring periods, and administered radiation doses. Cai et al. recently tried to comprehensively combine these results, however their analysis highlight some challenging point that requires further consideration, due to an evaluation limited to one time point after RAI [23].

Thus, considering the increasing number of younger individuals undergoing RAI for DTC, addressing potential reproductive concerns is essential. Therefore, this study aims to systematically evaluate available data to clarify the extent to which RAI affects gonadal function in male patients, ultimately contributing to a better understanding of its implications for reproductive health.

## Materials and methods

This systematic review was performed in line with the Meta-analysis of Observational Studies in Epidemiology (MOOSE) guidelines (Supplementary Fig. 1). The systematic literature review has been registered on the International Prospective Register for Systematic Reviews (PROSPERO ID 1009825).

### Literature search

The literature search was performed using the following words ‘radioactive iodine’, ‘iodine’, I-131, ‘sperm’, ‘semen’ and ‘fertility, combined with MESH terms AND/OR.

The literature search was performed until February 28th, 2025 independently by two authors (D.S., G.S.), and conflicts were resolved by a third investigator (F.P.), evaluating only English-language articles including human participants. The Endnote software (Version X9.2, Clarivate Analytics (US) LLC) was used for literature management and duplication filtration and removal.

### Study selection

The literature search was performed considering the following inclusion criteria: (i) prospective and retrospective design, (ii) in which men (iii) with DTC (iv) were treated with RAI after surgery, (v) reporting follicle-stimulating hormone (FSH) serum levels both before and after RAI.

Case reports were excluded. No other exclusion criteria were considered. Similarly, not only observational studies were extracted, but all trials design were considered.

### Outcome and quality assessment

The primary outcome was FSH serum levels evaluated before (pre-RAI) and after (post-RAI) RAI administration, considering the time lapses since treatment and the mean I-131 dosage used.

Other outcomes extracted from each study, when available, were: patients’ age, luteinizing hormone (LH) serum levels and semen analysis parameters (i.e. sperm concentration, progressive sperm motility and morphology).

All data were extracted as mean and standard deviation or converted in, when reported differently.

### Statistical analysis

Descriptive analyses were conducted and the characteristics of the enrolled patients and the results obtained after RAI administration were described, considering the time between the treatment and the evaluation.

The Newcastle-Ottawa Scale (NOS) was used to evaluate study’s related risk of bias.

A meta-analysis was conducted using random-effects models, with effect size evaluated through the non-standardized mean difference. The Hedges estimator was applied for inference, and the Knapp-Hartung adjustment was used for standard error calculation. Heterogeneity was assessed using I^2^ statistics. Even when low heterogeneity was detected, a random-effect model was applied because the validity of heterogeneity tests can be limited with a small number of component studies. Data heterogeneity exploration and possible outliers identification were performed using the Galbraith graph inspection. This analysis was completed by the ‘leave-one-out’ sensitivity analysis, removing each study one at a time to evaluate its influence on the overall estimate.

To assess the robustness of the findings and explore potential sources of heterogeneity, sensitivity analyses were performed. First, heterogeneity and possible outliers were evaluated through visual inspection of Galbraith plots. Trials identified as outliers were subsequently excluded, and the meta-analysis was repeated to determine whether the main findings were consistent. In addition, a leave-one-out sensitivity analysis was conducted, whereby the meta-analysis was iteratively recalculated after removing each study in turn, to assess the influence of individual studies on the overall effect estimates.

A meta-regression analysis was first performed using FSH as the independent variable and patients’ age as a dependent one, applying the random-effects model with Hedges’ analysis and standard error adaptation using the Knapp-Hartung method.

All analyses were performed using the “Statistical Package for the Social Science” software for Windows (version 28.0; SPSS Inc, Chicago, IL). Statistical significance was considered for p values < 0.05.

## Results

**Fig. 1:**
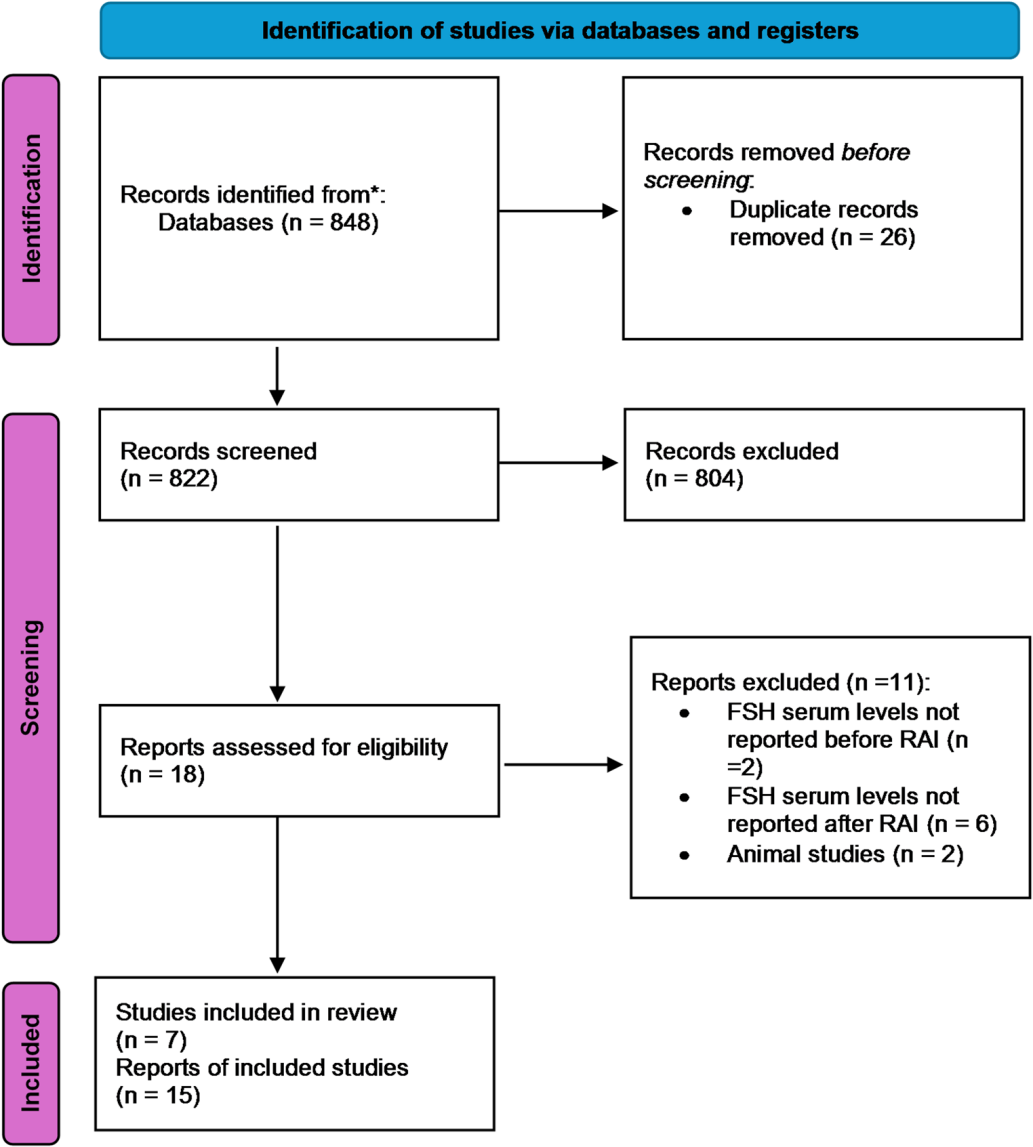
PRISMA checklist. RAI = radioactive iodine]

The literature search obtained 822 studies, of which 18 studies [27–43] were selected for full-text examination (Fig. 1). Among these studies, seven were finally included in the analysis according to inclusion and exclusion criteria (Fig. 1). Considering these manuscripts, data were extracted for 15 trials for a total of 460 men evaluated.

Table 1 summarizes data from studies included in the analysis. The included studies were published between 1994 and 2023 and conducted across various regions of the world in tertiary centers: five in Europe (two in Italy, one in the United Kingdom, one in France, and one in Germany), one in the Middle East (Iran), and one in South America (Brazil). The study design was prospective in five studies and retrospective in the remaining two. The included studies reported varying follow-up intervals: two studies assessed outcomes at three months post-RAI, four at six months, six at twelve months, and one at eighteen months. The sample size ranged from 18 to 122 patients, with three studies including more than 50 participants. The mean age of the treated group ranged from 32 to 49 years. The mean radioiodine dosage administered ranged from 100 to 346 mCi.

**Table 1:** Studies included in the analysis. Data are reported as mean *±* standard deviation. Radio-iodine doses are reported in each original study as mean value, without information about standard deviation or variance

				Control Group						Post-RAI Group							
Author	Year	Journal	RAI reason	*N*	Age (years)	FSH (IU/L)	LH (IU/L)	Sperm concentration (million/mL)	Progressive motility (%)	*N*	Age (years)	FSH (IU/L)	LH (IU/L)	Sperm concentration (million/mL)	Progressive motility (%)	RAI mean dosage (mCi)	Time frame interval of observations (months)
Pacini	1994	J Nuclear Med	DTC	103	35.3 ± 18.3	6.5 ± 3.1	NA	NA	NA	103	36.8 ± 15.2	15.3 ± 9.9	NA	NA	NA	167.0	12
Canale	2015	Clin Endocrinol	DTC	20	30.8 ± 5.4	5.2 ± 1.2	3.0 ± 0.2	54.5 ± 7.1	12.7 ± 3.9	20	32.1 ± 3.5	7.9 ± 1.2	4.1 ± 0.4	27.9 ± 6.5	10.8 ± 2.1	345.7	6, 12
Hyer	2002	Clin Endocrinol	DTC	122	35.0 ± 1.4	7.1 ± 1.1	NA	NA	NA	122	35.0 ± 1.4	6.1 ± 0.9	NA	NA	NA	100.0	12
Soltani	2023	Int J Reprod BioMed	DTC	18	35.6 ± 9.7	12.5 ± 1.7	5.0 ± 1.3	38.2 ± 14.4	42.0 ± 12.2	8	35.8 ± 9.9	13.3 ± 1.5	5.4 ± 1.0	34.3 ± 18.4	41.1 ± 13.0	150.0	3, 12
Bourcigaux	2018	Hum Reprod	DTC	24	35.6 ± 5.5	3.2 ± 2.2	3.7 ± 2.3	82.1 ± 108.7	50 ± 15.8	24	35.6 ± 5.5	6.9 ± 3.2	3.7 ± 2.1	36.7 ± 36.2	45.5 ± 11.8	100.0	3, 6
Rosario	2006	Thyroid	DTC	52	45.0 ± 9.6	6.8 ± 1.2	3.2 ± 0.8	NA	NA	52	45.0 ± 9.6	16.8 ± 2.6	4.8 ± 1.2	NA	NA	121.6	6, 12
Wichers	2020	Eur J Nucl Med	DTC	25	49.0 ± 5.1	5.4 ± 0.8	2.8 ± 0.3	NA	NA	25	49.0 ± 5.1	21.3 ± 2.4	5.9 ± 0.7	NA	NA	300.0	6, 12, 18

*DTC = differentiated thyroid cancer; FSH = follicle-stimulating hormone; LH = luteinizing hormone; NA = not available; RAI = radioactive iodine]*

Table 2 reported the risk of biases of studies included in the analysis.

**Table 2:** Newcastle-Ottawa scale (NOS) evaluating study-related risk of bias

Study	Selection (0–4)	Comparability (0–2)	Outcome (0–3)	Total Score (0–9)	Quality Rating
Bourcigaux et al., 2018	3	1	2	6	Moderate
*Canale et al. 2025*	*4*	*1*	*2*	*7*	Moderate
*Hyetr et al. 2002*	*2*	*1*	*2*	*5*	Moderate
*Pacini et al. 1994*	*3*	*1*	*2*	*6*	Moderate
*Rosario et al. 2006*	*3*	*2*	*3*	*8*	Moderate
*Soltani et al. 2023*	*3*	*1*	*2*	*6*	Moderate
*Wichers et al. 2000*	*1*	*1*	*3*	*5*	Moderate

### Hormones evaluation

**Fig. 2:**
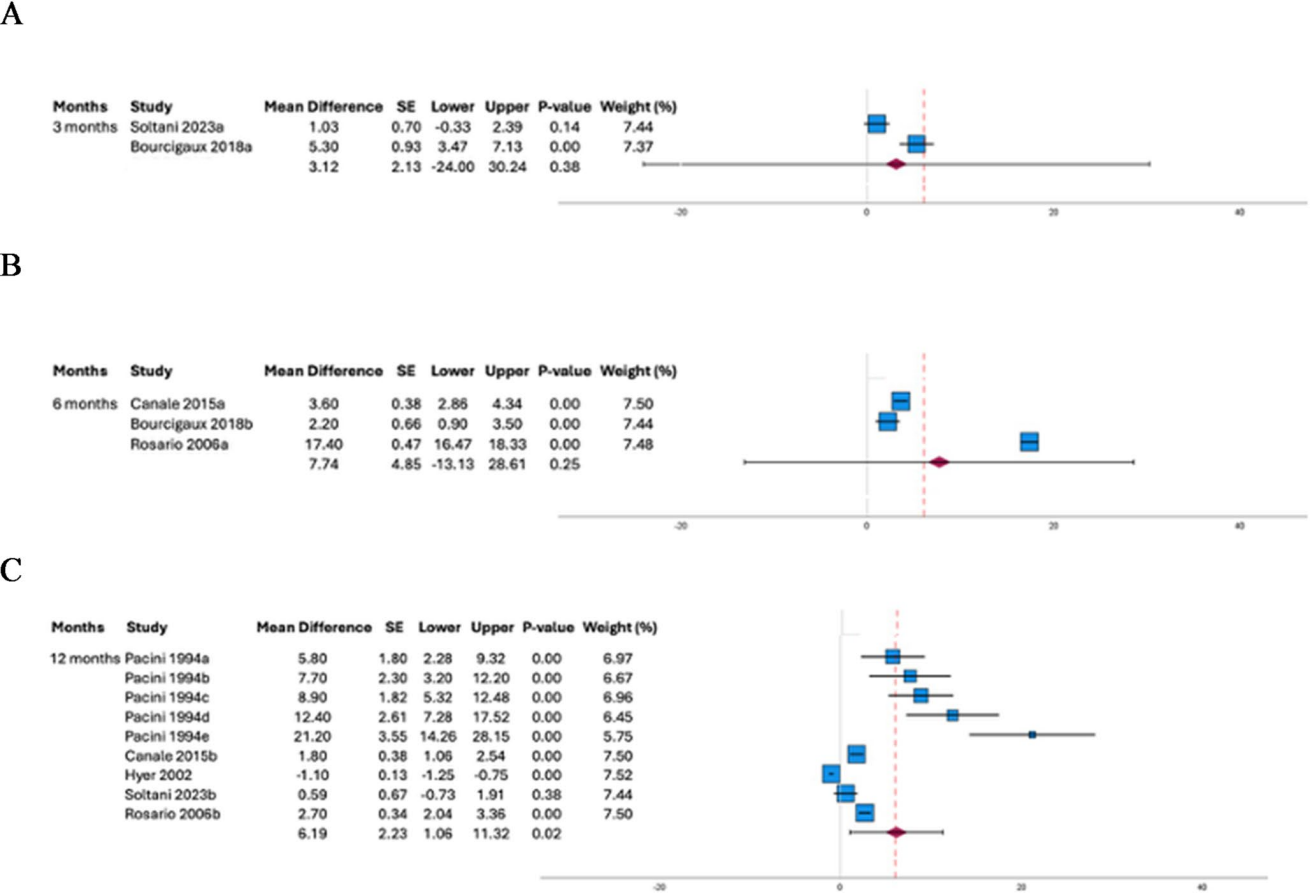
Forest plot showing follicle-stimulating hormone (FSH) serum levels (IU/L) comparing pre- and post- radioiodine administration. Panel A shows the comparison at 3 months, panel B at 6 months and panel C at 12 months of radioiodine administration

FSH serum levels were significantly higher after RAI administration after 12 months of RAI administration (*n* = 307 patients, *p* = 0.002) (Fig. 2). Otherwise, FSH serum levels did not change after RAI at 3 (*n* = 32 patients, *p* = 0.380) and 6 months (*n* = 121 patients, *p* = 0.090, respectively) (Fig. 2). Mean FSH serum levels were 7.1 ± 2.1 IU/L at baseline and 13.7 ± 6.7 IU/L 12 months after RAI.

**Fig. 3:**
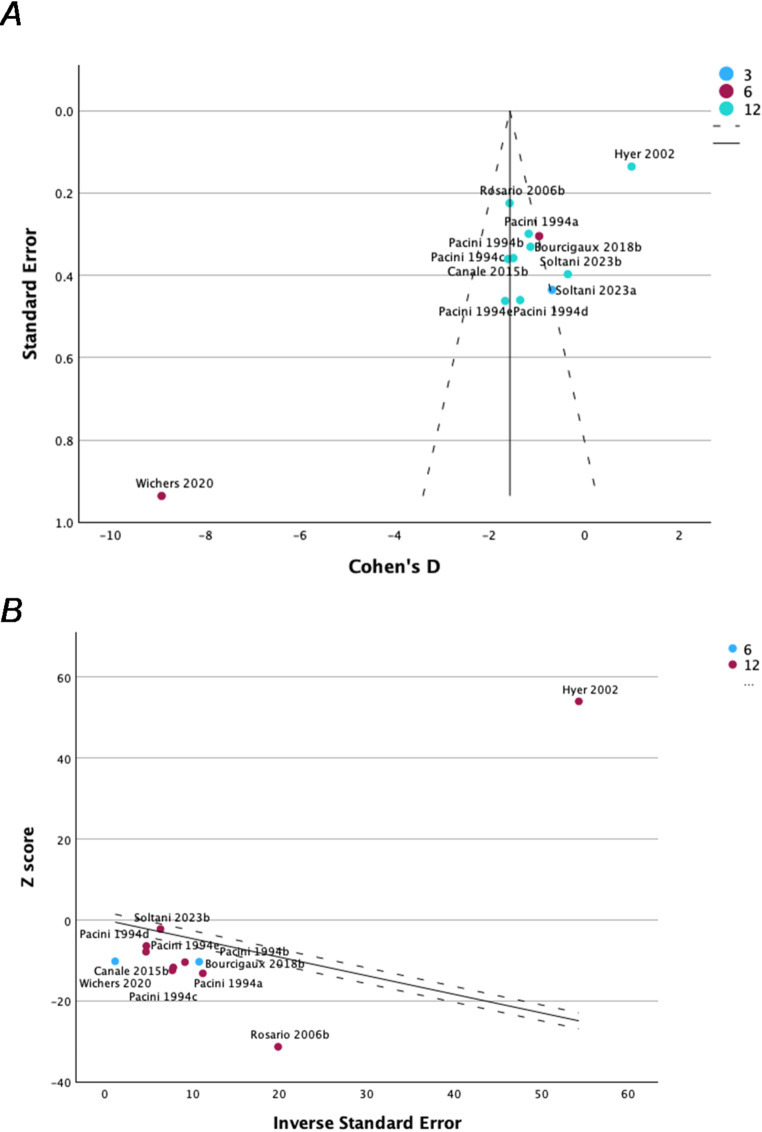
Outliers search evaluated with both funel plot (Panel A) and Galbraith graph (Panel B)

Considering the high heterogeneity obtained (I^2^ 99%, *p* < 0.001), data heterogeneity and possible outliers presence was evaluated using the Galbraith graph inspection (Fig. 3). This analysis highlighted five trials from four studies [32, 36, 40, 43], that have been removed in subsequent meta-analysis. This sensitivity analysis confirmed the FSH serum levels increase after RAI administration at 12 months of follow-up (mean difference 8.47 IU/L, SE 2.64 95%CI: 1.67–15.26 IU/L, *p* = 0.020) (Supplementary Fig. 2). However, this analysis was not able to completely adjust the heterogeneity among studies and or the existence of potential publication biases. A leave-one-out sensitivity analysis was subsequently performed, confirming the significant increase in FSH levels 12 months after RAI treatment, regardless of which study was excluded. Notably, this analysis identified the study by Hyer et al. [32] as having the greatest influence in attenuating the observed FSH increase. When this study was removed, the effect size of the FSH increase was higher than in the other sub-analyses (effect size = 1.33, SE = 0.20, *p* < 0.001).

In the group of studies evaluating the effects of RAI at 12 months, one publication resulted in five different trials. Specifically, Pacini et al. included distinct patient groups treated with different RAI doses, which were originally considered as five separate datasets. However, each of these trials alone could represent a source of bias due to the disproportionate weight it carries in the analysis. Indeed, the publication includes five different studies with the same authors, methodology, and potential biases, within a meta-analysis of relatively few studies and subjects. To address this potential confounder, we re-ran the meta-analysis including only one dataset from Pacini et al. [37], selecting the subgroup with an RAI dose closest to the average used across the other studies. This revised analysis showed no statistically significant change in FSH levels after RAI (Hedges’ G = 0.76, SE = 0.51, 95% CI: − 0.26 to 1.77, *p* = 0.140). However, the leave-one-out sensitivity analysis indicated that the FSH increase at 12 months remained significant when the study by Hyer et al. [32] was excluded (Hedges’ G = 1.26, SE = 0.27, 95% CI: 0.73 to 1.73, *p* < 0.001), confirming the robustness of the observed effect.

Meta-regression analysis was performed considering FSH as dependent variable and the patients’ age as dependent one. FSH was directly related to patients’ age (Chi-squared 72.8, *p* < 0.001), as expected (Supplementary Fig. 3). Also removing the two trials with mean age clearly shifted to the right (above 40 years), the regression analysis confirmed the increasing FSH with increasing age (Chi-squared 103.1, *p* < 0.001).

Meta-regression analysis was performed using the mean FSH difference between pre- and post- RAI administration as independent variable and the RAI dose used as dependent one, showing a significant increasing trend. However, since RAI dosages were reported as mean and standard deviation in only one study, while in the other studies they were extracted as maximum doses, this analysis should be interpreted with caution.

LH serum levels were reported in nine trials, from five different studies [29, 36, 39, 40, 43]. LH serum levels were not significantly different comparing pre- and post-treatment at 3 (mean difference 0.5 IU/L, SE 0.2 IU/L, 95%CI: −2.4, 3.4 IU/L, *p* = 0.280), 6 (mean difference 1.6 IU/L, SE 1.0 IU/L, 95%CI: −2.9, 6.1 IU/L, *p* = 0.268) and 12 months (mean difference 0.4 IU/L, SE 0.4 IU/L, 95%CI: −1.5, 2.2 IU/L, *p* = 0.483) after RAI administration (Supplementary Fig. 4).

### Semen analysis

Only six trials from three studies reported semen analysis among study endpoints [29, 39, 40]. Sperm concentration was not significantly different at 3 (mean difference − 29.8 million/mL, SE 27.4 million/mL, 95%CI: −378.5, 319.0 million/mL, *p* = 0.474), and 12 months (mean difference − 15.3 million/mL, SE 12.9 million/mL, 95%CI: −179.1, 148.5 million/mL, *p* = 0.446) after RAI administration (Supplementary Fig. 5). However, it was significantly lower at 6 months the meta-analysis although only two studies reported the data (mean difference − 25.7 million/mL, SE 0.3 million/mL, 95%CI: −29.8, −21.6 million/mL, *p* = 0.010) (Supplementary Fig. 5). Similarly, also progressive sperm motility was not significantly different at 3 (mean difference − 2.1%, SE 1.4%, 95%CI: −20.6, 16.3%, *p* = 0.382), 6 (mean difference − 0.8%, SE 0.3%, 95%CI: −4.5, 2.8%, *p* = 0.212) and 12 months (mean difference − 2.8%, SE 0.2%, 95%CI: −6.0, 0.3%, *p* = 0.055).

## Discussion

This study systematically evaluated available data suggesting a significant increase in FSH serum levels following RAI administration for DTC management in men. Notably, this elevation is delayed, as it is not observed at 3- or 6-month post-treatment but becomes apparent at 12 months. Additionally, the increase in FSH appears to be dose-dependent on I-131 administrated. These findings suggest that RAI may negatively impact male fertility, although critical challenges in this field must be carefully considered. Indeed, beyond the inherent limitations of the meta-analytic approach, our analysis highlights significant constraints that may negatively affect the generalization of the findings.

Radioiodine is efficiently concentrated in differentiated follicular thyroid cells, due to presence of the sodium/iodide symporter (NIS). However, NIS transcript was also detected in Leydig cells of human testes despite a ten-fold lower concentration [44]. Presence of NIS in germ cells has not been elucidated, but there is evidence of NIS expression in germ cells tumors (seminomas and embryonal carcinomas) [45]. Pendrin, an anion exchange protein, was found to be expressed in Sertoli cells [46]. Although their exact physiological role is unclear, it is known that iodine excess in rats is associated with testosterone reduction through inhibition of steroidogenesis [47] and there is also evidence of an inverse correlation between urinary iodine and circulating testosterone in men [48]. Nonetheless, due to expression of these iodine transport channels, testes can be considered iodine-concentrating organs, although with a reduced NIS expression. Thus, radioiodine is likely to concentrate both in the interstitial and seminiferous compartments of the testis. Hyer et al., reported a relatively low median estimated radiation dose after RAI exposure (between ~ 6.4–21.2 cGy after a 3–21.2.2 GBq exposure) [32]. Despite this predicted exposure level is unlikely to impact significantly on Leydig cell function, accumulation in Sertoli cell could impact more heavily on germ cells. With this in mind, we could speculate that RAI could have a gonadotoxic effect on the testis, as well as for other radiations. Indeed, several researches suggested varying susceptibility of different testicular cell types to X-ray, with germ cell populations being the most sensitive [49, 50]. Radiation could impact spermatogonia, which are highly sensitive cells due to their mitotic activity, and spermatids, which lack DNA damage repair proteins after their post meiotic differentiation. Spermatocytes, on the other hand, might remain mildly affected and able to further differentiate into spermatozoa. This different radiation sensibility of spermatogenic cells could explain why the negative effect of radiation on semen parameters is generally delayed [51]. Thus, the observed increase in FSH serum levels after RAI administration suggests either direct or indirect impact of I-131 on testicular function. However, this effect appears to be delayed, as it is not evident in the first few months following RAI administration but becomes noticeable at the one-year follow-up. The interpretation of this trend is challenging. Theoretically, FSH levels rise when damage affects spermatogonial function. Therefore, one would expect an immediate increase in serum FSH levels following RAI, with spermatogenic failure appearing later. However, the lack of long-term follow-up limits our ability to assess whether this effect persists, worsens, or resolves over time. Furthermore, we cannot determine how long I-131 remains in the body and testes, nor its precise impact on the survival of spermatogenic cell populations. Thus, we can only speculate that the testes are sensitive to I-131, which may enter and accumulate in the testicular parenchyma, suggesting a potential depot-effect. The hypothesis could consider the physiological pituitary FSH production and secretion, which is primarily regulated by inhibin B, secreted by Sertoli cells under gonadotropin stimulation itself. We could speculate that in the first months after RAI, Sertoli cells continue to produce inhibin B, maintaining a normal hypothalamic-pituitary-gonadal axis and preventing FSH alterations. However, after this initial “latency period,” the damage becomes apparent, requiring increased FSH stimulation to sustain normal sperm production. A sustained FSH rise implies impaired Sertoli cell function or germ cell loss, reducing inhibin B feedback. As a consequence, at 12 months post-RAI, semen parameters remain unchanged, but at the cost of elevated FSH serum levels, necessary to maintain spermatogenesis. Interestingly, the rise in FSH levels following I-131 administration is clearly dose-dependent, indicating greater damage to the seminiferous tubules with increasing I-131 dosages. However, none of the studies included have been able to assess the exact amount of I-131 that may accumulate in the testicular compartment. The distribution of RAI in the human body primarily occurs in thyroid tissue but can also accumulate in other tissues, including the stomach, salivary glands, mammary glands (especially during lactation), lacrimal glands, nasal mucosa, and placenta [52–54]. Despite these observations, the lack of specific data on I-131 accumulation in human testicular tissue remains a major limitation in fully understanding the gonadotoxic effects of I-131 treatment.

The rise in FSH serum levels detected by these analyses suggests a compensatory mechanism, indicating a reduction in sperm production, both quantitatively and qualitatively. Here, we are not able to demonstrate a sperm quantity reduction after RAI, but the results raise the question whether RAI administration negatively affects spermatogenesis quality. However, the current literature lacks data on the modification of sperm quality parameters other than “standard” semen parameters. Sperm DNA fragmentation and chromatin integrity is a known semen qualitative indicator that has shown interesting associations with recurrent miscarriage and infertility [55]. Its evaluation after RAI could highlight spermatogenesis impairment even in presence of near-normal semen parameters, but so far chromatin integrity investigation after I-131 administration is episodic [56]. However, the hypothesis that RAI administration affects sperm production (at least from a qualitative point of view) is further supported by the absence of LH changes after RAI administration, suggesting that Leydig cell function is largely unaffected and that the observed effect directly involves the testis, specifically the spermatogenic epithelium. However, this result is not confirmed considering the lack of testosterone measurement in these settings. Moreover, is this FSH increase merely a compensatory mechanism, or does it signal progressive damage to sperm production that will become more evident over time? Our meta-analysis, along with the existing literature, remains insufficient and fragmented, preventing definitive conclusions.

Another critical consideration of the reliability of our results is the role of age in the observed increase in FSH serum levels. Our analysis demonstrates that FSH levels correlate with patients’ age, raising the possibility that age-related factors may contribute to these findings. However, the exact influence of age remains unclear. Age-related declines in testicular reserve may amplify RAI effects, warranting stratified analyses in future studies. However, our finding of FSH elevation at 12 months contrasts with previous reports indicating peak FSH levels at 3–6 months [23]. This discrepancy may be attributed to both study heterogeneity and a dose-response relationship. Regarding study heterogeneity, one key factor is the variability in RAI doses, which ranged from an average of 100.0 mCi to more than 550 mCi across studies. Additionally, follow-up durations varied, and not all studies in the meta-analysis reported FSH serum levels at 3-, 6-, and 12-months post-RAI. As for the dose-response relationship, meta-regression analysis confirms that higher cumulative RAI doses are associated with prolonged FSH elevation and potential permanent germinal damage, particularly in patients receiving multiple treatments. These factors represent significant sources of bias in the included studies. However, we cannot rule out the possibility of publication bias. Various statistical approaches used to assess this bias indicate a high risk, which could not be fully mitigated by sensitivity analysis. Furthermore, the interpretation of our findings is constrained by the limited number of available studies, reducing the statistical power of sensitivity analyses. Additional subgrouping of included studies would further limit the amount of data available for analysis. Moreover, these results were obtained without control groups, restricting causal inferences. An additional limitation of our study is the lack of information regarding patients’ remission status after RAI therapy, which may have influenced both treatment response and subsequent gonadal outcomes. Moreover, data on pre-treatment TSH levels were not available across the included studies. Since TSH represents a relevant parameter potentially associated with the impact of RAI therapy on gonadal function, its absence limits our ability to fully assess this relationship. Finally, selection bias in the included studies cannot be excluded. Specifically, the enrollment of patients with metastatic or recurrent disease—who more frequently require multiple RAI doses—may lead to an overestimation of potential harm.

The lack of extensive, high-quality research raises concerns about the reliability of our conclusions and underscores the need for further studies. To accurately assess the impact of RAI administration on male fertility, future research should incorporate larger cohorts, extended follow-up periods, and more rigorous methodologies to minimize bias and enhance data reliability. While our findings mathematically suggest that RAI administration may lead to a delayed increase in FSH levels, the clinical significance of this change remains uncertain. The potential effects on male reproductive health require further investigation to determine the true safety implications of RAI therapy. To address these uncertainties, specifically designed studies are needed. These should involve prospective controlled cohorts comparing RAI-treated patients with non-RAI DTC cohorts, standardized dosimetry, and follow-up periods exceeding 12 months.
